# Total Synthesis and Pharmacological Investigation of Cordyheptapeptide A

**DOI:** 10.3390/molecules22060682

**Published:** 2017-05-27

**Authors:** Suresh Kumar, Rajiv Dahiya, Sukhbir Lal Khokra, Rita Mourya, Suresh V. Chennupati, Sandeep Maharaj

**Affiliations:** 1 Institute of Pharmaceutical Sciences, Kurukshetra University, Kurukshetra 136119, Haryana, India; sureshmpharma@rediffmail.com (S.K.); slkhokra@kuk.ac.in (S.L.K.); 2 Laboratory of Peptide Research and Development, School of Pharmacy, Faculty of Medical Sciences, The University of the West Indies, St. Augustine, Trinidad & Tobago, West Indies; Sandeep.Maharaj@sta.uwi.edu; 3 School of Pharmacy, College of Medicine and Health Sciences, University of Gondar, P.O. Box 196, Gondar 6200, Ethiopia; ritz_pharma@yahoo.co.in; 4 Department of Pharmacy, College of Medical and Health Sciences, Wollega University, P.O. Box 395, Nekemte, Ethiopia; sureshchennupati@rediffmail.com

**Keywords:** cordyheptapeptide A, solution-phase peptide synthesis, coupling, cytotoxicity, macrocyclization, biological activity, insect pathogenic fungus

## Abstract

The present investigation reports the synthesis of a phenylalanine-rich *N*-methylated cyclopeptide, cordyheptapeptide A (**8**), previously isolated from the insect pathogenic fungus *Cordyceps* sp. BCC 1788, accomplished through the coupling of *N*-methylated tetrapeptide and tripeptide fragments followed by cyclization of the linear heptapeptide unit. Structure elucidation of the newly synthesized cyclopolypeptide was performed by means of FT-IR, ^1^H-NMR, ^13^C-NMR, and fast atom bombardment mass spectrometry (FABMS), and screened for its antibacterial, antidermatophytic, and cytotoxic potential. According to the antimicrobial activity results, the newly synthesized *N*-Methylated cyclopeptide exhibited potent antibacterial activity against Gram-negative bacteria *Pseudomonas aeruginosa* and *Klebsiella pneumoniae* and antifungal activity against dermatophytes *Trichophyton mentagrophytes* and *Microsporum audouinii* at a concentration of 6 μg/mL, in comparison to the reference drugs, gatifloxacin and griseofulvin. In addition, cyclopolypeptide **8** displayed suitable levels of cytotoxicity against *Dalton’s lymphoma ascites* (DLA) and *Ehrlich’s ascites carcinoma* (EAC) cell lines.

## 1. Introduction

Nature is an attractive source of new therapeutic compounds. In drug discovery, microorganisms play a tremendous role especially by producing a number of candidates that are effective against microbes [1]. The previous literature is enriched with potential data indicating the ability of microorganisms such as fungi and bacteria, which produce a variety of natural products with diverse bioactivities [2,3]. Currently, microorganism-derived peptides (MdPs) are attracting the attention of scientists [4,5] because of the unique molecular structures and a wide array of associated bioactivities like antimycobacterial and antimalarial activity [6,7], antibacterial activity [8,9], cytotoxicity [10], and antiviral activity [11]. Cyclopeptides produced by microbes including cycloaspeptides, halolitoralins [12], psychrophilins, talaromins, ustiloxins, and unguisins are associated with many biological properties and some of the peptides from fungi have even gained entrance into the pharmaceutical market. e.g., cyclosporins, ergopeptides, etc.

The structure of a homodectic heptapeptide, cordyheptapeptide A, previously isolated from the insect pathogenic fungus *Cordyceps* sp. BCC 1788, was confirmed by spectroscopic techniques including FT-IR, ^1^H/^13^C-NMR, distortionless enhancement of polarisation transfer (DEPT), high-resolution electron ionization mass spectrometry (HREIMS) and the amino acid residues were established on the basis of correlation spectroscopy (COSY), total correlation spectroscopy (TOCSY), heteronuclear multiple quantum correlation (HMQC), and heteronuclear multiple bond correlation (HMBC) spectroscopy. In terms of the biological response, natural cordyheptapeptide A exhibited antimalarial activity against *Plasmodium falciparum* K1 and cytotoxicity to Vero cell lines with 50% inhibitory concentration values of 5.35 and >56.88 μM, respectively [13]. Another peptide of the series, cordyheptapeptide B, was isolated from a fungal strain *Cordyceps* sp. and the absolute configuration of the cordyheptapeptide B was indicated by chromatographic analysis of the acid hydrolyzate. Cordyheptapeptide B was found to exhibit cytotoxicity against several cell lines including KB, BC, NCI-H187, and Vero cell lines with IC_50_ values in range of 0.66–3.1 μM [14]. In 2011, the total synthesis of cordyheptapeptide B was reported for the first time utilizing a solution-phase technique [15]. Cordyheptapeptide B differs from cordyheptapeptide A by having an *N*-methyl-l-phenylalanine residue instead of the *N*-methyl-l-tyrosine. Three new cyclic peptides, cordyheptapeptides C–E, were isolated from the marine-derived fungus *A. persicinum* SCSIO 115 and their planar structures were confirmed by extensive mass spectrometry (MS), 1D and 2D NMR spectroscopic analyses. Cordyheptapeptides C and E displayed cytotoxicity against the SF-268, MCF-7, and NCI-H460 tumor cell lines with IC_50_ values in the range of 2.5 to 12.1 μM [16].

Given the diverse pharmacological activities possessed by MdPs and other cyclooligopeptides [17,18,19] and the continued efforts of our research group [15,20,21,22,23,24,25,26,27,28,29,30,31,32,33,34,35,36,37,38,39,40,41,42,43,44,45] for synthesizing *N*-methylated and other cyclopolypeptides for quantitative yield in the laboratory, the present work was directed toward the synthesis, characterization, and bioactivity screening of a phenylalanine-rich cyclopolypeptide, cordyheptapeptide A for the antimicrobial and cytotoxic properties.

## 2. Results

### 2.1. Chemistry

**Figure 1:**
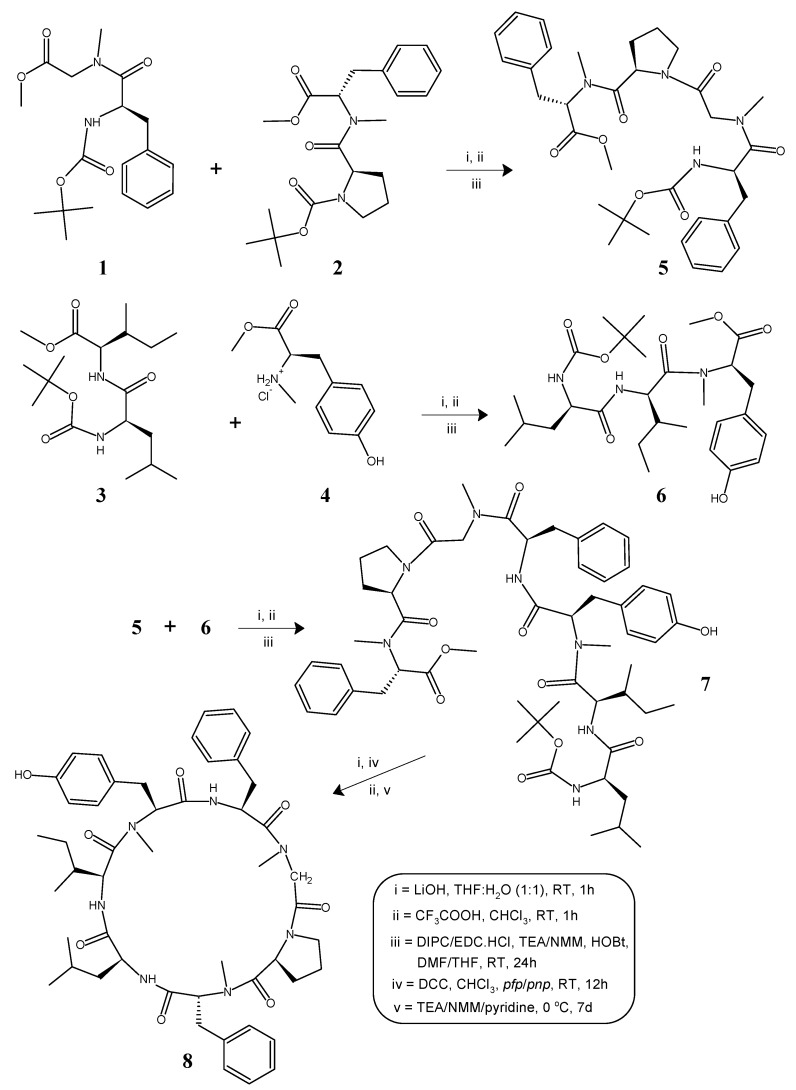
Synthetic route of the *N*-methylated cyclopeptide, cordyheptapeptide A (**8**).

The cycloheptapeptide molecule was split into three dipeptide units viz. Boc-l-Phe-N(*Me*)Gly-OMe (**1**), Boc-l-Pro-d-N(*Me*)Phe-OMe (**2**), and Boc-l-Leu-l-Ile-OMe (**3**), and a single amino acid unit l-N(*Me*)Tyr-OMe·HCl (**4**). *N*-methylation of Boc-protected phenylalanine and tyrosine methyl esters was done by treatment with methyliodide and sodium hydride as per the literature [46]. Dipeptide units (**1**–**3**) were prepared by the coupling of Boc-amino acids such as Boc-l-Phe, Boc-l-Pro, and Boc-l-Leu with the corresponding amino acid methyl ester hydrochlorides such as N(*Me*)Gly-OMe·HCl, d-N(*Me*)Phe-OMe·HCl, and l-Ile-OMe·HCl by following the modified Bodanzsky method [47]. After deprotection at the carboxy terminus, dipeptide **1** was coupled with dipeptide **2**, deprotected at the amino terminus, to obtain the tetrapeptide unit Boc-l-Phe-N(*Me*)Gly-l-Pro-d-N(*Me*)Phe-OMe (**5**). The carboxyl group of dipeptide **3** was deprotected by alkaline hydrolysis using lithium hydroxide (LiOH) and the deprotected peptide was coupled with the amino acid unit **4**, utilizing different carbodiimides to obtain the tripeptide unit Boc-l-Leu-l-Ile-l-N(*Me*)Tyr-OMe (**6**). After removal of the ester group of tripeptide **6** and the Boc-group of tetrapeptide **5**, the deprotected units (**5a**, **6a**) were coupled to obtain the linear heptapeptide unit Boc-l-Leu-l-Ile-l-N(*Me*)Tyr-l-Phe-N(*Me*)Gly-l-Pro-d-N(*Me*)Phe-OMe (**7**). The methyl ester group of the linear peptide fragment was replaced by the pentafluorophenyl (*pfp*)/*p*-nitrophenyl (*pnp*) ester group. The Boc-group of the resulting compound was removed using trifluoroacetic acid (CF_3_COOH), and the deprotected linear fragment was now cyclized by keeping the entire contents at 0 °C for 7 days in the presence of catalytic amounts of triethylamine (TEA) or *N*-methylmorpholine (NMM) or pyridine to get the cyclic product **8**. The structures of the newly synthesized cycloheptapeptide as well as that of the intermediate tri/tetra/heptapeptides were confirmed by FT-IR, ^1^H-NMR spectroscopy, and elemental analysis. In addition, the mass spectra and ^13^C-NMR spectroscopy were recorded for the linear and cyclic heptapeptide only. The synthetic pathway for the newly synthesized *N*-methylated heptacyclopeptide is shown in Figure 1.

### 2.2. Pharmacological Activity Studies

The synthesized linear and cycloheptapeptide (**7**, **8**) were subjected to the short term in vitro cytotoxicity study against the *Dalton’s lymphoma ascites* and *Ehrlich’s ascites carcinoma* cell lines at concentrations of 62.5–3.91 μg/mL using 5-fluorouracil (5-FU) as the reference compound. The activity was determined by measuring the inhibition (%) of the cell lines [48]. The CTC_50_ values were determined by the graphical extrapolation method. The results of the cytotoxic activity studies are presented in Table 1.

Moreover, the linear and heptacyclopeptide (**7**, **8**) were further evaluated for their antimicrobial activity against the Gram-positive bacteria *Bacillus subtilis* and *Staphylococcus aureus*, and Gram-negative bacteria *Pseudomonas aeruginosa*, *Klebsiella pneumoniae*, dermatophytes *Microsporum audouinii*, *Trichophyton mentagrophytes*, diamorphic fungi *Candida albicans*, and other fungal strains, including *Aspergillus niger* at concentrations of 50–6.25 μg/mL by using the modified Kirby-Bauer disc diffusion method [49]. The minimum inhibitory concentration (MIC) values of the test compounds were determined by the tube dilution technique. Gatifloxacin and griseofulvin were used as the reference drugs and DMF/DMSO were used as the control. The results of the antibacterial and antifungal studies are presented in Table 2.

## 3. Discussion

**Figure 2:**
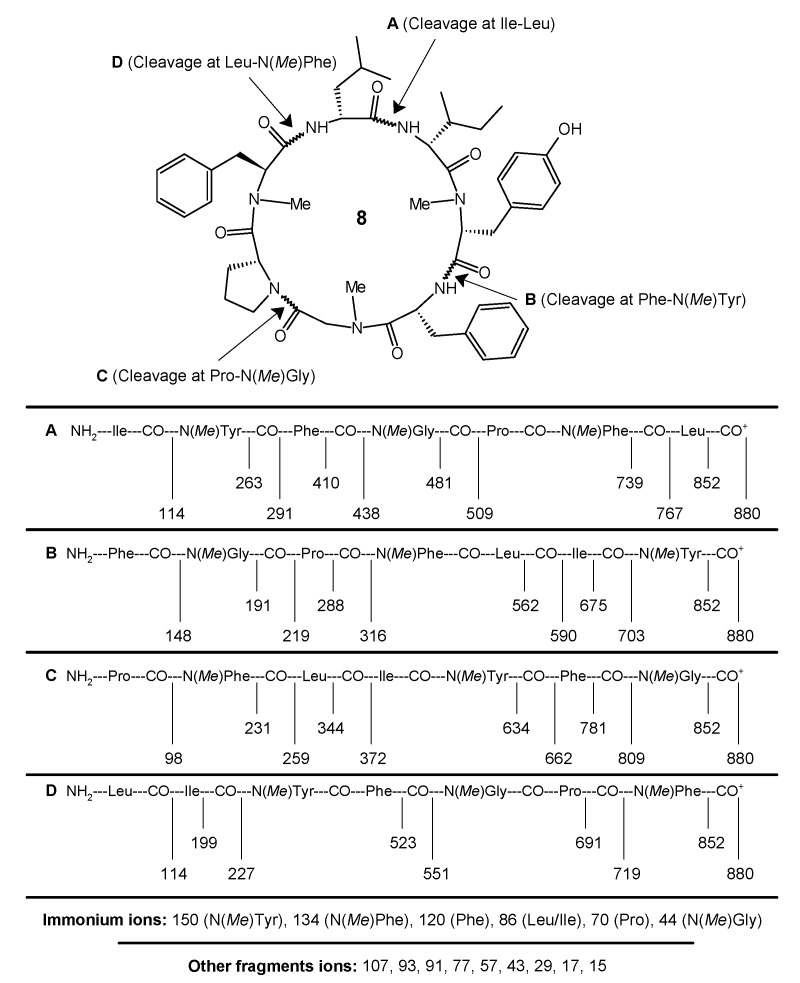
Mass fragmentation pattern for the newly synthesized *N*-methylated cyclopeptide, cordyheptapeptide A (**8**).

The synthesis of the *N*-methylated cyclopolypeptide **8** was accomplished with 83% yield, and the *N*-methylmorpholine proved to be an effective base for the cyclization of the linear heptapeptide unit. The cyclization of the linear peptide fragment was supported by the disappearance of the absorption bands at 1744, 1268, 1393, 1381, and 939 cm^−1^ (C=O_str_, C–O_str_, ester and C–H_bend_, CH_3(rock)_, *tert*-butyl group) in the IR spectra of **8**. The formation of the cyclopeptide was further confirmed by the disappearance of the singlets at 3.56 and 1.51 ppm corresponding to the three protons of the methyl ester group and the nine protons of the *tert*-butyl group of the Boc in the ^1^H-NMR spectrum and the disappearance of the singlets at 154.5 and 79.8, and 52.9 and 28.3 ppm corresponding to the carbon atoms of the ester and *tert*-butyl groups in the ^13^C-NMR spectrum of **8**, respectively. Furthermore, the ^1^H-NMR and ^13^C-NMR spectra of the synthesized cyclic heptapeptide showed the characteristic peaks confirming the presence of all of the 65 protons and 49 carbon atoms. A large ^13^C chemical shift difference in the ^13^C chemical shifts between the Cβ and Cγ signals of the proline residue (Δδβγ = 32.0 − 23.6 = 8.4) indicates a *cis* configuration for the proline residue in the synthesized *N*-methylated cyclopolypeptide **8**. The retention times (*t*_R_, min) of the observed peaks of the 1-fluoro-2-4-dinitrophenyl-5-l-alanine amide (FDAA) derivatized hydrolysis products of cyclopeptide **8** were 18.27 for the amino acid gly, 27.23 for l-leu, 28.90 for l-ile, 20.54 for l-tyr, 17.44 for l-phe, 22.36 for d-phe, and 17.42 for l-pro, and were all in agreement with the values for standard amino acid derivatives during the HPLC analysis. The appearance of the pseudomolecular ion peak (M + 1)^+^ at *m*/*z* = 880 corresponding to the molecular formula C_49_H_65_N_7_O_8_ in the mass spectrum of **8**, along with other fragment ion peaks resulting from the cleavage at ‘Phe-N(*Me*)Tyr’, ‘Pro-N(*Me*)Gly’, ‘Ile-Leu’, and ‘Leu-N(*Me*)Phe’ amide bonds showed the exact sequence of the attachment of all of the seven amino acid moieties in a chain (Figure 2). In addition, the presence of the immonium ion peaks at *m*/*z* 150 [N(*Me*)Tyr], 134 [N(*Me*)Phe], 120 [Phe], 86 [Leu/Ile], 70 [Pro], and 44 [N(*Me*)Gly] further confirmed all the amino acid moieties in the cyclopeptide structure. Furthermore, the elemental analysis of **8** afforded the values with a tolerance of ±0.02 strictly in accordance with the molecular composition.

Comparison of the antibacterial activity data suggested that the cycloheptapeptide **8** possessed potent bioactivity against the Gram-negative bacteria *K. pneumonia* and *P. aeruginosa*, in addition to a satisfactory level of activity against the Cutaneous fungi *M. audouinii* and *T. mentagrophytes* with the MIC values of 6 μg/mL when compared to the reference drugs, gatifloxacin and griseofulvin. No significant level of bioactivity was observed against the pathogenic fungi *C. albicans* and *A. niger* and the Gram-positive bacteria *B. subtilis* and *S. aureus*, in comparison to the standard drugs. Moreover, the newly synthesized *N*-methylated cyclopeptide **8** exhibited a good level of cytotoxic activity against the DLA and EAC cell lines with CTC_50_ values of 10.6 and 14.6 μM, respectively, in comparison to the standard drug, 5-fluorouracil (5-FU) (CTC_50_ values of 37.4 and 90.6 μM, respectively). The possible mechanism of the cytotoxic action of cycloheptapeptide **8** might be through apoptosis via induction of the early cell death, nuclear fragmentation, and the internucleosomal DNA scission. In addition, the analysis of the pharmacological activity data revealed that cycloheptapeptide **8** displayed more bioactivity against the pathogenic microbes and cell lines when compared to its linear form **7**. The enhanced activity is due to the reduction in the degree of freedom for each constituent within the ring by the cyclization of the peptides. Usually, cyclic peptides show better biological activity compared to their linear counterparts due to the conformational rigidity, which decreases the entropy term of the Gibbs free energy, therefore allowing the enhanced binding toward target molecules, or receptor selectivity. Another advantage from the cyclic structure is the resistance to hydrolysis by the exopeptidases due to the lack of both amino and carboxyl termini. Furthermore, the cyclic peptides can be resistant even to the endopeptidases, as the structure is less flexible than the linear peptides [50]. Cordyheptapeptide A differs from the cordyheptapeptide B in one amino acid moiety i.e., *N*-methyl-l-tyrosine in place of the *N*-methyl-l-phenylalanine residue. This structural change results in the enhanced bioactivity against the Gram-negative bacteria and variation in the cytotoxic properties against the DLA and EAC cell lines [14].

## 4. Materials and Methods

Amino acids, trifluoroacetic acid, pentafluorophenol (*pfp*), *p*-nitrophenol (*pnp*), pyridine (C_5_H_5_N), *N*-methylmorpholine (NMM), triethylamine (TEA), Diisopropylcarbodiimide (DIPC), Dicyclohexylcarbodiimide (DCC), 1-ethyl-3-(3-dimethylaminopropyl)carbodiimide hydrochloride (EDC·HCl), and di-*tert*-butyl-pyrocarbonate (Boc_2_O) were purchased from Spectrochem Limited (Mumbai, India). The IR spectra were recorded on a Shimadzu 8700 FTIR spectrophotometer (Shimadzu, Japan) using a thin film supported on KBr pellets for the synthesized cyclic heptapeptide and CHCl_3_ as the solvent for the intermediate semisolids. The ^1^H-NMR and ^13^C-NMR spectra were recorded on a Bruker AC-NMR spectrometer (Brucker, Billerica, MA, USA) at 300 MHz using CDCl_3_ as the solvent and tetramethylsilane (TMS) as the internal standard. The mass spectrum was recorded on a JMS-DX 303 Mass spectrometer (Jeol, Tokyo, Japan) operating at 70 eV using the fast atom bombardment technique. 

### 4.1. General Method for the Synthesis of Linear N-methylated Tri/Tetrapeptide Units *(**5**, **6**)*

The *N*-methyl amino acid methyl ester hydrochloride or dipeptide methyl ester (0.01 mol) was dissolved in DMF (25 mL). To this, TEA (0.021 mol) was added at 0 °C and the reaction mixture was stirred for 15 min. The Boc-dipeptide (0.01 mol) in the DMF (25 mL), DIPC (1.26 g, 0.01 mol), and HOBt (1.34 g, 0.01 mol) were added with stirring. The stirring was first done for 1 h at 0–5 °C and then for a further 24 h at room temperature (RT). After the completion of the reaction, the reaction mixture was diluted with an equal amount of water. The precipitated solid/semisolid was then filtered/separated and washed with water, and purified from the mixture of CHCl_3_-petroleum ether (b.p. 60–80 °C) followed by cooling at 0 °C to obtain the title compounds.

#### 4.1.1. *Tert*-Butyloxycarbonyl-l-phenylalanyl-N(*Me*)glycyl-l-prolyl-d-N(*Me*)phenylalanine Methyl Ester (**5**)

Semisolid mass; Yield 79%; [α]_D_ = −52.3° (*c* = 0.25, CHCl_3_); R*_f_* = 0.48 (CHCl_3_·MeOH—8:2); IR (CHCl_3_): *v* 3136 (m, -NH str, amide), 2999, 2996 (m, -CH str, cyclic CH_2_, Pro), 2927, 2622, 2854–2851 (m, -CH str, asym and sym, CH_2_), 2870, 2866 (m, -CH str, sym, CH_3_), 1742 (s, -C=O str, ester), 1664–1659, 1642 (s, -C=O str, 3° and 2° amide), 1587, 1479 (m, skeletal bands, arom. rings), 1532 (m, -NH bend, 2° amide), 1391, 1376 (s, -CH bend, *tert*-Butyl group), 1270 (s, C-O str, ester), 933 (w, CH_3_ rocking, *tert*-Butyl group), 726–722, 690, 687 (s, -CH bend, out-of-plane, arom. ring) cm^−1^; ^1^H-NMR (300 MHz, CDCl_3_): δ 7.52–7.48 (2H, tt, *J* = 6.8, 4.45 Hz, *m*-H’s, Phe-1), 7.15–7.12 (1H, t, *J* = 6.2 Hz, *p*-H, Phe-2), 7.05–7.01 (2H, tt, *J* = 6.75, 4.5 Hz, *m*-H’s, Phe-2), 6.93–6.90 (1H, t, *J* = 6.25 Hz, *p*-H, Phe-1), 6.86–6.84 (2H, dd, *J* = 8.8, 4.15 Hz, *o*-H’s, Phe-1), 6.75–6.73 (2H, dd, *J* = 8.75, 4.2 Hz, *o*-H’s, Phe-2), 6.51 (1H, br. s, -NH, Phe-1), 5.39–5.36 (1H, t, *J* = 5.2 Hz, α-H, Phe-2), 4.87–4.83 (1H, q, *J* = 5.55 Hz, α-H, Phe-1), 4.45–4.42 (1H, t, *J* = 6.9 Hz, α-H, Pro), 3.89 (2H, s, α-H, Gly), 3.59–3.56 (2H, t, *J* = 7.15 Hz, δ-H’s, Pro), 3.54 (3H, s, OCH_3_), 3.17–3.11 (4H, m, β-H’s, Phe-1 and Phe-2), 2.99 (3H, s, *N*CH_3_, Phe), 2.94 (3H, s, *N*CH_3_, Gly), 2.69–2.64 (2H, q, β-H’s, Pro), 1.96–1.89 (2H, m, γ-H’s, Pro), 1.52 (9H, s, *tert*-Butyl group) ppm; C_33_H_44_N_4_O_7_ (608): calcd. C 65.11, H 7.29, N 9.20; found C 65.10, H 7.32, N 9.22.

#### 4.1.2. *Tert*-Butyloxycarbonyl-l-leucyl-l-isoleucyl-l-N(*Me*)tyrosine Methyl Ester (**6**)

Semisolid mass; Yield 83%; [α]_D_ = +81.3° (*c* = 0.25, CHCl_3_); R*_f_* = 0.71 (CHCl_3_·MeOH - 9:1); IR (CHCl_3_): *v* 3372 (m, -OH str, arom. ring), 3136, 3132 (m, -NH str, amide), 2929, 2854 (m, -CH str, asym and sym, CH_2_), 2967–2963, 2869, 2863 (m, -CH str, asym and sym, CH_3_), 1745 (s, -C=O str, ester), 1667, 1644–1641 (s, -C=O str, 3° and 2° amide), 1588, 1471 (m, skeletal bands, arom. ring), 1539, 1535 (m, -NH bend, 2° amide), 1466 (m, -CH bend (scissoring), CH_2_), 1393, 1375 (s, -CH bend, *tert*-Butyl group), 1379, 1364 (s, -CH bend, *iso*-propyl group), 1274 (s, C-O str, ester), 933, 920 (w, CH_3_ rocking, *tert*-Butyl and *iso*-propyl groups), 729, 685 (s, -CH bend, oop, arom. ring) cm^−1^; ^1^H-NMR (300 MHz, CDCl_3_): δ 7.09 (1H, br. s, -NH, Ile), 6.87–6.83 (2H, dd, *J* = 8.6, 4.85 Hz, *m*-H’s, Tyr), 6.79–6.75 (2H, dd, *J* = 8.55, 5.25 Hz, *o*-H’s, Tyr), 6.06 (1H, br. s, -NH, Leu), 5.95 (1H, br. s, -OH, Tyr), 4.46–4.43 (1H, t, *J* = 5.8 Hz, α-H, Tyr), 4.37–4.34 (1H, t, *J* = 8.6 Hz, α-H, Ile), 4.22–4.18 (1H, q, *J* = 6.85 Hz, α-H, Leu), 3.57 (3H, s, OCH_3_), 3.11–3.09 (2H, d, *J* = 5.8 Hz, β-H’s, Tyr), 3.04 (3H, s, *N*CH_3_), 2.06–1.95 (3H, m, β-H’s, Leu and Ile), 1.65–1.59 (2H, m, γ-H’s, Ile), 1.58–1.53 (1H, m, γ-H’s, Leu), 1.50 (9H, s, *tert*-Butyl group), 1.04–1.02 (3H, d, *J* = 5.9 Hz, γ′-H’s, Ile), 1.01–0.99 (6H, d, *J* = 6.25 Hz, δ-H’s, Leu), 0.95–0.93 (3H, d, *J* = 7.75 Hz, δ-H’s, Ile) ppm; C_28_H_45_N_3_O_7_ (535): calcd. C 62.78, H 8.47, N 7.84; found C 62.75, H 8.49, N 7.85.

### 4.2. Deprotection of the Tetrapeptide Unit *(**5**)* at the Amino End and Tripeptide Unit *(**6**)* at the Carboxyl End

The Boc-protected tetrapeptide **5** (6.08 g, 0.01 mol) was dissolved in CHCl_3_ (15 mL) and treated with CF_3_COOH (2.28 g, 0.02 mol). The resulting solution was stirred at room temperature for 1 h, and then washed with a saturated NaHCO_3_ solution (25 mL). The organic layer was dried over anhydrous Na_2_SO_4_ and concentrated under reduced pressure. The crude product was purified by the crystallization from CHCl_3_ and petroleum ether (b.p. 40–60 °C) to obtain the pure deprotected compound **5a**.

For deprotection at the carboxyl end, LiOH (0.36 g, 0.015 mol) was added to a solution of tripeptide **6** (5.35 g, 0.01 mol) in THF·H_2_O (1:1, 36 mL) at 0 °C. The mixture was stirred at room temperature for 1 h and then acidified to pH 3.5 with 1 M H_2_SO_4_. The aqueous layer was extracted with Et_2_O (3 × 25 mL). The combined organic extracts were dried over anhydrous Na_2_SO_4_ and concentrated under reduced pressure. The crude product was finally crystallized from methanol and ether to obtain the pure deprotected compound **6a**.

#### 4.2.1. l-Phenylalanyl-N(*Me*)glycyl-l-prolyl-d-N(*Me*)phenylalanine Methyl Ester (**5a**)

Semisolid mass; Yield 83%; [α]_D_ = –84.1° (*c* = 0.25, CHCl_3_); R*_f_* = 0.61 (CHCl_3_·MeOH - 8:2); IR (CHCl_3_): *v* 3496, 3389 (w, -NH str, amine), 2997, 2995 (m, -CH str, cyclic CH_2_, Pro), 2926–2622, 2855–2849 (m, -CH str, asym and sym, CH_2_), 2868, 2865 (m, -CH str, sym, CH_3_), 1739 (s, -C=O str, ester), 1667–1662, 1643 (s, -C=O str, 3° and 2° amide), 1616 (m, -NH bend, amine), 1589, 1477 (m, skeletal bands, arom. rings), 1534 (m, -NH bend, 2° amide), 1334 (m, -CN str, amine), 1269 (s, C-O str, ester), 725–722, 689, 685 (s, -CH bend, out-of-plane, arom. ring) cm^−1^; ^1^H-NMR (300 MHz, CDCl_3_): δ 7.29–7.25 (2H, tt, *J* = 6.75, 4.5 Hz, *m*-H’s, Phe-1), 7.17–7.14 (1H, t, *J* = 6.15 Hz, *p*-H, Phe-2), 7.06–7.02 (2H, tt, *J* = 6.8, 4.45 Hz, *m*-H’s, Phe-2), 6.97–6.94 (1H, t, *J* = 6.2 Hz, *p*-H, Phe-1), 6.77–6.69 (4H, m, *o*-H’s, Phe-1 and Phe-2), 5.38–5.35 (1H, t, *J* = 5.15 Hz, α-H, Phe-2), 4.46–4.43 (1H, t, *J* = 6.85 Hz, α-H, Pro), 3.98–3.93 (1H, m, α-H, Phe-1), 3.82 (2H, s, α-H, Gly), 3.61–3.58 (2H, t, *J* = 7.2 Hz, δ-H’s, Pro), 3.52 (3H, s, OCH_3_), 3.07–3.05 (2H, d, *J* = 5.6 Hz, β-H’s, Phe-2), 2.97 (3H, s, *N*CH_3_, Phe-2), 2.92 (3H, s, *N*CH_3_, Gly), 2.75–2.73 (2H, d, *J* = 5.65 Hz, β-H’s, Phe-1), 2.68–2.63 (2H, q, β-H’s, Pro), 2.25 (2H, s, NH_2,_ Phe-1), 1.95–1.89 (2H, m, γ-H’s, Pro) ppm; C_28_H_36_N_4_O_5_ (508): calcd. C 66.12, H 7.13, N 11.02; found C 66.10, H 7.15, N 11.05.

#### 4.2.2. *Tert*-Butyloxycarbonyl-l-leucyl-l-isoleucyl-l-N(*Me*)tyrosine (**6a**)

Semisolid mass; Yield 79%; [α]_D_ = +67.7° (*c* = 0.25, CHCl_3_); R*_f_* = 0.82 (CHCl_3_·MeOH—9:1); IR (CHCl_3_): *v* 3315–2530 (m/br, -OH str, -COOH), 3375 (m, -OH str, Tyr), 3133, 3129 (m, -NH str, amide), 2927, 2856 (m, -CH str, asym and sym, CH_2_), 2966–2962, 2868–2863 (m, -CH str, asym and sym, CH_3_), 1710 (m, -C=O str, -COOH), 1668, 1644–1639 (s, -C=O str, 3° and 2° amide), 1589, 1469 (m, skeletal bands, arom. ring), 1536–1532 (m, -NH bend, 2° amide), 1462 (m, -CH bend (scissoring), CH_2_), 1423 (m, C-O-H bend, -COOH), 1392, 1377 (s, -CH bend, *tert*-Butyl group), 1378, 1362 (s, -CH bend, *iso*-propyl group), 936, 919 (w, CH_3_ rocking, *tert*-Butyl and *iso*-propyl groups), 727, 683 (s, -CH bend, oop, arom. ring) cm^−1^; ^1^H-NMR (300 MHz, CDCl_3_): δ 8.19 (2H, br. s, -OH, Tyr and -CO*OH*), 7.08 (1H, br. s, -NH, Ile), 6.82–6.78 (2H, dd, *J* = 8.55, 4.9 Hz, *m*-H’s, Tyr), 6.93–6.89 (2H, dd, *J* = 8.6, 5.3 Hz, *o*-H’s, Tyr), 6.02 (1H, br. s, -NH, Leu), 5.35–5.32 (1H, t, *J* = 8.55 Hz, α-H, Ile), 4.52–4.49 (1H, t, *J* = 5.75 Hz, α-H, Tyr), 4.19–4.15 (1H, q, *J* = 6.9 Hz, α-H, Leu), 3.17 (3H, s, *N*CH_3_), 3.13–3.11 (2H, d, *J* = 5.75 Hz, β-H’s, Tyr), 2.05–1.96 (3H, m, β-H’s, Leu and Ile), 1.67–1.61 (2H, m, γ-H’s, Ile), 1.59–1.54 (1H, m, γ-H’s, Leu), 1.52 (9H, s, *tert*-Butyl group), 1.03–1.01 (3H, d, *J* = 5.85 Hz, γ′-H’s, Ile), 0.99–0.97 (6H, d, *J* = 6.3 Hz, δ-H’s, Leu), 0.93–0.91 (3H, d, *J* = 7.8 Hz, δ-H’s, Ile) ppm; C_27_H_43_N_3_O_7_ (521): calcd. C 62.17, H 8.31, N 8.06; found C 62.19, H 8.32, N 8.09.

### 4.3. Procedure for the Preparation of the Linear Heptapeptide Unit and Its Cyclized Form *(**7**, **8**)*

The tetrapeptide methyl ester, l-Phe-N(*Me*)Gly-l-Pro-d-N(*Me*)Phe-OMe (**5a**, 5.08 g, 0.01 mol) was dissolved in 30 mL of THF, 0.021 mol of NMM was added at 0 °C, and the resulting mixture was stirred for 15 min. The Boc-protected tripeptide, Boc-l-Leu-l-Ile-l-N(*Me*)Tyr-OH (**6a**, 5.21 g, 0.01 mol) was dissolved in 30 mL of THF and DIPC/EDC·HCl (1.26 g/1.92 g, 0.01 mol) and HOBt (1.34 g, 0.01 mol) were added to the above mixture with stirring. The stirring was continued for 24 h, after which the reaction mixture was filtered and the filtrate was washed with 25 mL each of the 5% NaHCO_3_ and saturated NaCl solutions. The organic layer was dried over the anhydrous Na_2_SO_4_, filtered, and evaporated in vacuum. Finally, the recrystallization of the crude product was carried out from a mixture of the CHCl_3_-petroleum ether (b.p. 40–60 °C) followed by cooling at 0 °C to obtain the Boc-l-Leu-l-Ile-l-N(*Me*)Tyr-l-Phe-N(*Me*)Gly-l-Pro-d-N(*Me*)Phe-OMe (**7**) as the pale-yellow semisolid mass. The linear heptapeptide unit (**7**, 5.06 g, 0.005 mol) was then deprotected at the carboxyl terminal using the lithium hydroxide (LiOH, 0.18 g, 0.0075 mol) to obtain Boc-l-Leu-l-Ile-l-N(*Me*)Tyr-l-Phe-N(*Me*)Gly-l-Pro-d-N(*Me*)Phe-OH. To a solution of the deprotected heptapeptide (4.99 g, 0.005 mol) in CHCl_3_ (50 mL), pentafluorophenol (*pfp*, 1.23 g, 0.0067 mol) and DCC (1.06 g, 0.005 mol) were added followed by stirring at room temperature for 12 h. The filtrate of the above reaction mixture was washed with 10% NaHCO_3_ (3 × 20 mL) and 5% HCl (2 × 20 mL) solutions to obtain the corresponding pentafluorophenyl ester Boc-l-Leu-l-Ile-l-N(*Me*)Tyr-l-Phe-N(*Me*)Gly-l-Pro-d-N(*Me*)Phe-O*pfp*. The Boc-group of the resulting unit (4.66 g, 0.004 mol) was removed using CF_3_COOH (0.91 g, 0.008 mol) to obtain the deprotected product, l-Leu-l-Ile-l-N(*Me*)Tyr-l-Phe-N(*Me*)Gly-l-Pro-d-N(*Me*)Phe-O*pfp*, which was dissolved in CHCl_3_ (25 mL) and TEA/NMM/pyridine (2.8 mL/2.21 mL/1.61 mL, 0.021 mol) was added. Then, the entire contents were kept at 0 °C for 7 days. Then the reaction mixture was washed with 10% NaHCO_3_ (3 × 25 mL) and 5% HCl (2 × 25 mL) solutions. The organic layer was dried over anhydrous Na_2_SO_4_ and the crude cyclized compound was recrystallized from the CH_2_Cl_2_/*n*-hexane to obtain the cyclic product *cyclo*(l-leucyl-l-isoleucyl-l-N(*Me*)tyrosyl-l-phenylalanyl-N(*Me*)glycyl-l-prolyl-d-N(*Me*)phenylalanyl) (**8**).

#### 4.3.1. *Tert*-Butyloxycarbonyl-l-leucyl-l-isoleucyl-l-N(*Me*)tyrosyl-l-phenylalanyl-N(*Me*)glycyl-l-prolyl-d-N(*Me*)phenylalanine Methyl Ester (**7**)

Semisolid mass; Yield 78%; [α]_D_ = −103.2° (*c* = 0.25, MeOH); R*_f_* = 0.73 (CHCl_3_·MeOH—9:1); IR (CHCl_3_): *v* 3369 (m, -OH str, arom. ring), 3135–3129 (m, -NH str, amide), 2998–2994 (m, -CH str, cyclic CH_2_, Pro), 2928, 2625, 2853–2849 (m, -CH str, asym and sym, CH_2_), 2968, 2962, 2872, 2866 (m, -CH str, asym and sym, CH_3_), 1744 (s, -C=O str, ester), 1666–1659, 1643–1639 (s, -C=O str, 3° and 2° amide), 1589–1584, 1477–1473 (m, skeletal bands, arom. rings), 1537–1533 (m, -NH bend, 2° amide), 1393, 1381 (s, -CH bend, *tert*-Butyl group), 1375, 1364 (s, -CH bend, *iso*-propyl group), 1268 (s, C-O str, ester), 939, 921 (w, CH_3_ rocking, *tert*-Butyl and *iso*-propyl groups), 728–723, 689, 685 (s, -CH bend, out-of-plane, arom. rings) cm^−1^; ^1^H-NMR (300 MHz, CDCl_3_): δ 9.22 (1H, br. s, -NH, Phe-1), 7.19–7.16 (2H, tt, *J* = 6.75, 4.45 Hz, *m*-H’s, Phe-1), 7.14–7.11 (1H, t, *J* = 6.15 Hz, *p*-H, Phe-2), 7.08 (1H, br. s, -NH, Ile), 7.06–7.03 (2H, tt, *J* = 6.8, 4.5 Hz, *m*-H’s, Phe-2), 7.02–6.97 (2H, dd, *J* = 8.55, 4.9 Hz, *m*-H’s, Tyr), 6.92–6.89 (1H, t, *J* = 6.3 Hz, *p*-H, Phe-1), 6.85–6.83 (2H, dd, *J* = 8.75, 4.15 Hz, *o*-H’s, Phe-1), 6.74–6.69 (2H, dd, *J* = 8.6, 5.25 Hz, *o*-H’s, Tyr), 6.75–6.73 (2H, dd, *J* = 8.8, 4.15 Hz, *o*-H’s, Phe-2), 6.05 (1H, br. s, -NH, Leu), 5.97 (1H, br. s, -OH, Tyr), 5.38–5.35 (1H, t, *J* = 5.15 Hz, α-H, Phe-2), 4.89–4.85 (1H, q, *J* = 5.6 Hz, α-H, Phe-1), 4.45–4.42 (1H, t, *J* = 8.55 Hz, α-H, Ile), 4.40–4.37 (1H, t, *J* = 6.85 Hz, α-H, Pro), 4.35–4.32 (1H, t, *J* = 5.75 Hz, α-H, Tyr), 4.20–4.16 (1H, q, *J* = 6.9 Hz, α-H, Leu), 3.91 (2H, s, α-H, Gly), 3.61–3.58 (2H, t, *J* = 7.2 Hz, δ-H’s, Pro), 3.56 (3H, s, OCH_3_), 3.11–3.07 (4H, m, β-H’s, Phe-1 and Phe-2), 3.04 (3H, s, *N*CH_3_, Tyr), 2.99 (3H, s, *N*CH_3_, Phe-2), 2.95 (3H, s, *N*CH_3_, Gly), 2.89–2.87 (2H, d, *J* = 5.75 Hz, β-H’s, Tyr), 2.70–2.65 (2H, q, β-H’s, Pro), 2.05–1.93 (3H, m, β-H’s, Leu and Ile), 1.91–1.86 (2H, m, γ-H’s, Pro), 1.67–1.61 (2H, m, γ-H’s, Ile), 1.58–1.53 (1H, m, γ-H’s, Leu), 1.51 (9H, s, *tert*-Butyl group), 1.07–1.05 (3H, d, *J* = 5.85 Hz, γ′-H’s, Ile), 1.03–1.01 (6H, d, *J* = 6.3 Hz, δ-H’s, Leu), 0.99–0.97 (3H, d, *J* = 7.8 Hz, δ-H’s, Ile) ppm; ^13^C-NMR (CDCl_3_): δ = 175.3 (C=O, Tyr), 172.6 (C=O, Leu), 170.5, 169.5, 167.3 (3 C, C=O, Phe-1, Ile and Phe-2), 166.9, 165.1 (2 C, C=O, Gly and Pro), 156.2 (*p*-C, Tyr), 154.5 (C=O, Boc), 139.2 (γ-C, Phe-2), 136.6 (γ-C, Phe-1), 130.8 (2 C, *o*-C’s, Tyr), 129.6 (2 C, *o*-C’s, Phe-2), 128.5 (2 C, *m*-C’s, Tyr), 128.1 (2 C, *o*-C’s, Phe-1), 127.4 (2 C, *m*-C’s, Phe-1), 126.9 (2 C, *m*-C’s, Phe-2), 126.5 (γ-C, Tyr), 126.1, 125.6 (2 C, γ-C’s, Phe-1 and Phe-2), 79.8 (α-C, Boc), 60.1, 57.7, 54.5 (3 C, α-C’s, Tyr, Phe-2 and Pro), 52.9 (O*C*H_3_), 51.3, 48.2 (2 C, α-C’s, Leu and Ile), 46.8, 45.3 (2 C, α-C, Gly and Phe-1), 39.9 (δ-C, Pro), 38.0, 37.2 (2 C, β-C’s, Leu and Phe-1), 36.2 (N*C*H_3_, Gly), 34.4, 33.6, 32.3 (3 C, β-C’s, Ile, Tyr and Phe-2), 31.5, 29.7 (2 C, *NC*H_3_, Phe-2 and Tyr), 29.2 (β-C, Pro), 28.3 (3 C, β-C’s, Boc), 26.6, 25.2 (2 C, γ-C’s, Pro and Ile), 24.4 (2 C, δ-C’s, Leu), 22.1 (γ-C, Leu), 17.4 (γ′-C, Leu), 10.1 (δ-C, Ile); C_55_H_77_N_7_O_11_ (1012): calcd. C 65.26, H 7.67, N 9.69; found C 65.25, H 7.65, N 9.72.

#### 4.3.2. *Cyclo*(l-leucyl-l-isoleucyl-l-N(*Me*)tyrosyl-l-phenylalanyl-N(*Me*)glycyl-l-prolyl-d-N(*Me*)phenylalanyl) (**8**)

Pale yellow crystals; m.p. 178–180 °C (179.6–179.8 °C for natural cordyheptapeptide A [13]); Yield 83% (NMM), 79% (C_5_H_5_N), 68% (TEA); [α]_D_ = −68.6° (*c* = 0.56, CHCl_3_) (–68.5° for natural cordyheptapeptide A [12]); R*_f_* = 0.59 (CHCl_3_·MeOH—9:1); IR (KBr): *v* 3365 (m, -OH str, arom. ring), 3133, 3129–3126 (m, -NH str, amide), 2999–2993 (m, -CH str, cyclic CH_2_, Pro), 2929–2624, 2855, 2849 (m, -CH str, asym and sym, CH_2_), 2969–2963, 2874, 2867 (m, -CH str, asym and sym, CH_3_), 1668–1662, 1644, 1632 (s, -C=O str, 3° and 2° amide), 1588–1582, 1479–1474 (m, skeletal bands, arom. rings), 1539–1534 (m, -NH bend, 2° amide), 1376, 1364 (s, -CH bend, *iso*-propyl group), 923 (w, CH_3_ rocking, *iso*-propyl group), 729–722, 686, 682 (s, -CH bend, out-of-plane, arom. rings) cm^−1^; ^1^H-NMR (300 MHz, CDCl_3_): δ 8.64 (1H, br. s, -NH, Phe), 8.23 (1H, br. s, -NH, Leu), 7.65 (2H, tt, *J* = 6.75, 4.5 Hz, *m*-H’s, Phe-2), 7.44 (2H, tt, *J* = 6.8, 4.45 Hz, *m*-H’s, Phe-1), 7.23 (1H, t, *J* = 6.15 Hz, *p*-H, Phe-2), 7.01 (2H, dd, *J* = 8.6, 4.85 Hz, *m*-H’s, Tyr), 6.85 (1H, t, *J* = 6.25 Hz, *p*-H, Phe-1), 6.71 (2H, dd, *J* = 8.8, 4.15 Hz, *o*-H’s, Phe-1), 6.46 (2H, dd, *J* = 8.55, 5.3 Hz, *o*-H’s, Tyr), 6.37 (2H, dd, *J* = 8.8, 4.15 Hz, *o*-H’s, Phe-2), 5.95 (1H, br. s, -OH, Tyr), 5.89 (1H, br. s, -NH, Ile), 5.59 (1H, t, *J* = 5.2 Hz, α-H, Phe-2), 5.44 (2H, s, α-H, Gly), 5.37 (1H, q, *J* = 5.55 Hz, α-H, Phe-1), 4.95 (1H, q, *J* = 6.85 Hz, α-H, Leu), 4.46 (1H, t, *J* = 8.6 Hz, α-H, Ile), 4.35 (1H, t, *J* = 6.85 Hz, α-H, Pro), 3.64 (2H, t, *J* = 7.15 Hz, δ-H’s, Pro), 3.45 (1H, t, *J* = 5.8 Hz, α-H, Tyr), 3.07 (3H, s, *N*CH_3_, Phe), 2.94 (3H, s, *N*CH_3_, Gly), 2.74 (2H, q, β-H’s, Pro), 2.62 (3H, s, *N*CH_3_, Tyr), 2.46 (2H, d, *J* = 5.8 Hz, β-H’s, Tyr), 2.13 (4H, m, β-H’s, Phe-1 and Phe-2), 1.75 (2H, m, γ-H’s, Pro), 1.61 (2H, m, γ-H’s, Ile), 1.47 (3H, m, β-H’s, Leu and Ile), 1.25 (6H, d, *J* = 6.25 Hz, δ-H’s, Leu), 1.13 (3H, d, *J* = 5.9 Hz, γ′-H’s, Ile), 0.97 (3H, d, *J* = 7.75 Hz, δ-H’s, Ile), 0.85 (1H, m, γ-H’s, Leu) ppm; ^13^C-NMR (CDCl_3_): δ = 174.4 (C=O, Leu), 172.5 (C=O, Pro), 170.7 (C=O, Ile), 170.4 (C=O, Tyr), 170.1, 168.7 (2 C, C=O, Phe and *N*MePhe), 168.2 (C=O, Gly), 155.9 (*p*-C, Tyr), 139.4 (γ-C, Phe-2), 137.3 (γ-C, Phe-1), 133.1 (γ-C, Tyr), 132.3 (2 C, *m*-C’s, Phe-1), 130.2 (2 C, *o*-C’s, Tyr), 129.8 (2 C, *m*-C’s, Tyr), 128.9 (2 C, *o*-C’s, Phe-1), 128.1 (2 C, *m*-C’s, Phe-2), 127.2 (2 C, *m*-C’s, Phe-1), 122.9, 122.2 (2 C, γ-C’s, Phe-2 and Phe-1), 68.9, 58.6, 57.9, 54.8 (4 C, α-C’s, Tyr, Ile, Pro and Phe-2), 50.6, 49.9, 47.3 (3 C, α-C’s, Gly, Phe-1 and Leu), 43.5 (δ-C, Pro), 42.9, 42.3 (2 C, β-C’s, Leu and Phe-1), 40.1 (*NC*H_3_, Tyr), 39.8, 36.9 (2 C, β-C’s, Tyr and Phe-2), 36.6 (β-C, Ile), 35.7 (*NC*H_3_, Phe), 32.0 (β-C, Pro), 30.3 (*NC*H_3_, Gly), 29.7, 24.9, 23.6 (3 C, γ-C’s, Leu, Ile and Pro), 22.0 (2 C, δ-C’s, Leu), 17.1 (γ′-C, Leu), 10.3 (δ-C, Ile); MS (FAB, 70 eV): *m*/*z* (%) = 880 (100) [M + 1]^+^, 852 (13) [880-CO]^+^, 809 (77) [Pro-*N*(Me)Phe-Leu-Ile-*N*(Me)Tyr-Phe]^+^, 781 (29) [809-CO]^+^, 767 (49) [Ile-*N*(Me)Tyr-Phe-*N*(Me)Gly-Pro-*N*(Me)Phe]^+^, 739 (21) [767-CO]^+^, 719 (59) [Leu-Ile-*N*(Me)Tyr-Phe-*N*(Me)Gly-Pro]^+^, 703 (39) [Phe-*N*(Me)Gly-Pro-*N*(Me)Phe-Leu-Ile]^+^, 691 (13) [719-CO]^+^, 675 (26) [703-CO]^+^, 662 (52) [Pro-*N*(Me)Phe-Leu-Ile-*N*(Me)Tyr]^+^, 634 (32) [662-CO]^+^, 606 (69) [Ile-*N*(Me)Tyr-Phe-*N*(Me)Gly-Pro]^+^, 590 (78) [Phe-*N*(Me)Gly-Pro-*N*(Me)Phe-Leu]^+^, 578 (17) [606-CO]^+^, 562 (24) [590-CO]^+^, 551 (54) [Leu-Ile-*N*(Me)Tyr-Phe]^+^, 523 (25) [551-CO]^+^, 509 (44) [Ile-*N*(Me)Tyr-Phe-*N*(Me)Gly]^+^, 481 (22) [509-CO]^+^, 438 (52) [Ile-*N*(Me)Tyr-Phe]^+^, 410 (19) [438-CO]^+^, 372 (44) [Pro-*N*(Me)Phe-Leu]^+^, 344 (25) [662-CO]^+^, 316 (66) [Phe-*N*(Me)Gly-Pro]^+^, 291 (41) [Ile-*N*(Me)Tyr]^+^, 288 (22) [316-CO]^+^, 263 (20) [291-CO]^+^, 259 (39) [Pro-*N*(Me)Phe]^+^, 231 (15) [662-CO]^+^, 227 (37) [Leu-Ile]^+^, 219 (46) [Phe-*N*(Me)Gly]^+^, 199 (21) [227-CO]^+^, 191 (17) [219-CO]^+^, 150 (22) [*N*(Me)Tyr immonium ion, C_9_H_12_NO]^+^, 148 (16) [Phe]^+^, 134 (26) [*N*(Me)Phe immonium ion, C_9_H_12_N]^+^, 120 (16) [Phe immonium ion, C_8_H_10_N]^+^, 114 (20) [Ile/Leu]^+^, 107 (27) [C_7_H_7_O]^+^, 98 (14) [Pro]^+^, 93 (19) [C_6_H_5_O]^+^, 91 (23) [C_7_H_7_]^+^, 86 (29) [Leu/Ile immonium ion, C_5_H_12_N]^+^, 77 (20) [C_6_H_5_]^+^, 70 (24) [Pro immonium ion, C_4_H_8_N]^+^, 57 (18) [C_4_H_9_]^+^, 44 (9) [*N*(Me)Gly immonium ion, C_2_H_6_N]^+^, 43 (13) [C_3_H_7_]^+^, 29 (11) [C_2_H_5_]^+^, 17 (10) [OH]^+^, 15 (16) [CH_3_]^+^; C_49_H_65_N_7_O_8_ (879): calcd. C 66.87, H 7.44, N 11.14; found C 61.85, H 7.47, N 11.15.

### 4.4. Preparation and Analysis of the Marfey Derivatives

About 0.5 mg of the synthesized cycloheptapeptide **8** was hydrolyzed by heating in 1 mL of 6 M HCl at 110 °C for 24 h. After cooling, the solution was evaporated to dryness and redissolved in 50 μL of water. Then 100 μL of a 1% *w*/*v* solution of FDAA (Marfey’s reagent) in acetone was added to the acid hydrolyzate solution (or to 50 μL of a 50 mM solution of the respective amino acid). After addition of 20 μL of the 1 M NaHCO_3_ solution, the mixture was incubated for 1 h at 40 °C. The reaction was stopped by the addition of 10 μL of 2 M HCl. Finally, the solvents were evaporated to dryness and the residue was dissolved in 1 mL of MeOH. An aliquot of this solution (20 μL for the cyclopeptide **8** and 10 μL for the standards) was analyzed by HPLC (Phenomenex Luna C18, 4.6 × 250 mm, 5 μm, solvents: (A) water + 0.05% TFA, (B) MeCN, linear gradient: 0 min 35% B, 30 min 45% B, 1 mL min^−1^, 25 °C). The retention times (min) of the FDAA amino acid derivatives used as the standards were as follows: gly (18.24), l-ile (28.92), d-ile (33.32), l-tyr (20.51), d-tyr (33.08), l-leu (27.25), d-leu (25.05), l-phe (17.41), d-phe (22.32), l-pro (17.45), and d-pro (18.39). Retention times (min) and relative peak area (%) of the observed peaks of the FDAA derivatized hydrolysis products of cyclopeptide **8** were as follows: gly (18.27, 2.39%), l-leu (27.23, 2.48%), l-ile (28.90, 3.67%), l-tyr (20.54, 5.24%), l-phe (17.44, 7.33%), d-phe (22.36, 6.26%), and l-pro (17.42, 7.82%).

### 4.5. Biological Evaluation Procedures

#### 4.5.1. Cytotoxic Screening

The linear and cyclic heptapeptides (**7**, **8**) were subjected to the short term in vitro cytotoxicity study at 62.5–3.91 μg/mL against the DLA and EAC cell lines using 5-fluorouracil (5-FU) as the reference compound. The different dilutions of both compounds ranging from 62.5–3.91 μg/mL were prepared in Dulbecco’s minimum essential medium and 0.1 mL of each diluted test compound was added to 0.1 mL of the DLA cells (1 × 10^6^ cells/mL) and EAC cells (1 × 10^6^ cells/mL). The resulting suspensions were incubated at 37 °C for 3 h followed by performing the tryphan blue dye test and the calculation of the growth inhibition (%). The CTC_50_ values were determined by the graphical extrapolation method. The controls were also tested at 62.5–3.91 μg/mL against both cell lines. The results of the cytotoxicity studies are listed in Table 1.

#### 4.5.2. Antimicrobial Screening

The newly synthesized linear and cyclic heptapeptides (**7**, **8**) were evaluated for their antibacterial and antifungal potential against two Gram-positive bacteria, *B. subtilis* and *S. aureus*, two Gram-negative bacteria, *P. aeruginosa* and *K. pneumoniae*, and the diamorphic fungal strain *C. albicans* and three other fungal strains, including *A. niger* and two Cutaneous fungal strains *M. audouinii* and *T. mentagrophytes* at the concentrations of 50–6.25 μg/mL. The MIC values of the test compounds were determined by the tube dilution technique. Both the linear and cyclic heptapeptides were dissolved separately to prepare a stock solution of 1 mg/mL using the DMF or DMSO. The stock solution was aseptically transferred and suitably diluted with the sterile broth medium to contain seven different concentrations of each test compound ranging from 200–3.1 μg/mL in the different test tubes. The inoculation of all the tubes was carried out with one of the test microbes. The process was repeated with different test bacteria/fungi and the different samples. The tubes inoculated with the bacterial cultures were incubated at 37 °C for 18 h and the fungal cultures were incubated at 37 °C for 48 h. Finally, the presence/absence of growth of the bacteria/fungi was observed. From these results, the MIC of each test compound was determined against each test bacterium/fungus. Gatifloxacin and griseofulvin were used as the reference drugs with the pure solvents (DMF and DMSO) as the negative controls for the antibacterial and antifungal studies, respectively. The Petri plates inoculated with the bacterial cultures were incubated at 37 °C for 18 h and those inoculated with the fungal cultures were incubated at 37 °C for 48 h. The diameters of the inhibition zones (in mm) were measured and the average diameters for the test samples were calculated in triplicate. The diameters obtained for the test samples were compared with that produced by the standard drug. The results of the antimicrobial studies are presented in Table 2.

The experimental details of the biological activity studies are described in our previously published reports [51,52,53,54,55]. As per the International Union of Pure and Applied Chemistry (IUPAC) rules, the *N*-methylated cycloheptapeptide **8** can be named as “3,15-Dibenzyl-9-(*sec*-butyl)-12-(4-hydroxybenzyl)-6-isobutyl-2,11,17-trimethylperhydropyrrolo [1,2-*a*][1,4,7,10,13,16,19]heptaazacyclohenicosine-1,4,7,10,13,16,19-heptaone”.

## 5. Conclusions

The first successful synthesis of a *N*-methylated cyclopeptide, cordyheptapeptide A (**8**), was accomplished in the present study, with reasonable yield *via* the coupling reactions utilizing different carbodiimides. The EDC·HCl/TEA coupling method proved to be yield-effective, in comparison to the method utilizing the DIPC/TEA or NMM, providing 9–10% extra yield. The pentafluorophenyl ester was shown to be better than the *p*-nitrophenyl ester, for the activation of the acid functionality of the linear heptapeptide unit. The NMM was found to be a good base for the intramolecular cyclization of the linear peptide fragment in comparison to the TEA or pyridine. The synthesized heptacyclopeptide displayed potent antibacterial activity especially against the Gram-negative bacteria, and effectiveness against the pathogenic dermatophtytes and the significant level of cytotoxicity. The Gram-negative bacteria were found to be more sensitive than the Gram-positive bacteria towards the newly synthesized peptide. On passing the toxicity tests, the *N*-methylated cyclopeptide **8** may prove to be a good candidate for clinical studies and can be a new antibacterial, anti-dermatophyte, and the anticancer drug of the future.

## Figures and Tables

**Table 1:** Cytotoxic activity data for the linear and cycloheptapeptide (**7**, **8**).

Compd.	Conc. (μg/mL)	*Dalton’s Lymphoma Ascites* (DLA) Cells				*Ehrlich’s Ascites Carcinoma* (EAC) Cells			
		Live Cells Counted	No. of Dead Cells	% GI ^a^	CTC_50_ (μM) ^b^	Live Cells Counted	No. of Dead Cells	% GI	CTC_50_ (μM)
**7**	62.50	0	38	100.0		0	28	100.0	
	31.25	11	27	71.05		8	20	71.43	
	15.63	21	17	44.74	19.9	17	11	39.29	22.6
	7.81	25	12	34.21		21	7	25.00	
	3.91	34	4	10.53		24	4	14.29	
**8**	62.50	0	38	100.0		0	28	100.0	
	31.25	7	31	81.58		4	24	85.71	
	15.63	15	23	60.53	10.6	11	17	60.71	14.6
	7.81	22	16	42.11		18	10	35.71	
	3.91	27	10	28.95		22	6	21.43	
**Control**	62.50	38	0	–		28	0	–	
	31.25	38	0	–		28	0	–	
	15.63	38	0	–	–	28	0	–	–
	7.81	38	0	–		28	0	–	
	3.91	38	0	–		28	0	–	
**Std.**	62.50	0	38	100.0		0	28	100.0	
**(5-FU)**	31.25	0	38	100.0		0	28	100.0	
	15.63	10	28	73.68	37.4	11	17	60.71	90.6
	7.81	13	25	65.79		19	9	32.14	
	3.91	22	16	42.11		23	5	17.86	

^a^ % growth inhibition (GI) = 100 − [{(Cell_total_ – Cell_dead_) × 100}/Cell_total_]; ^b^ CTC_50_ = cytotoxic concentration inhibiting 50% of growth.

**Table 2:** Antimicrobial evaluation data for the linear and cycloheptapeptide (**7**, **8**).

Compound	Diameter of the Zone of Inhibition (mm)							
	*Bacterial Strains*				*Fungal Strains*			
	*B. sub.*	*S. aur.*	*P. aeru.*	*K. pneu.*	*C. alb.*	*M. audo.*	*A. niger*	*T. menta.*
**7**	10(50)	–	21(6)	23(6)	12(25)	18(6)	–	16(6)
**8**	12(50)	10(25)	25(6)	26(6)	15(25)	22(6)	9(25)	20(6)
DMF ^†^	–	–	–	–				
DMSO ^‡^					–	–	–	–
Gatifloxacin	18(12.5) *	27(6)	23(6)	25(6)	–	–	–	–
Griseofulvin	–	–	–	–	20(6)	18(6)	20(12.5)	19(6)

*B. sub*.: Bacillus subtilis, *S. aur.*: Staphylococcus aureus, *P. aeru.*: *Pseudomonas aeruginosa*, *K. pneu.*: *Klebsiella pneumoniae*, *C. alb.*: Candida albicans, *M. audo.*: *Microsporum audouinii*, *A. niger*: *Aspergillus niger*, *T. menta.*: Trichophyon mentagrophytes; * Values in the bracket are the MIC values (μg/mL); ^†^ dimethylformamide—negative control for the antibacterial studies; ^‡^ dimethylsulfoxide—negative control for the antifungal studies.

## Supplementary Materials

The following are available online. Figure S1: Mass spectrum for the *N*-methylated cyclic heptapeptide, cordyheptapeptide A (**8**), Figure S2: ^13^C-NMR spectrum for the *N*-methylated cyclic heptapeptide, cordyheptapeptide A (**8**), Table S1: Various steric and lipophilicity parameters for the *N*-methylated linear and cyclic heptapeptide (**7**, **8**).
