# Supplementary material for: Total Synthesis and Pharmacological Investigation of Cordyheptapeptide A
Source: Molecules. 2017 May 27;22(6):682. doi: 10.3390/molecules22060682 (PMC6152760; doi:10.3390/molecules22060682)
Supplement: Supplementary file 1 [file molecules-22-00682-s001.pdf]

*Supporting Information*

**Total Synthesis and Pharmacological Investigation of Cordyheptapeptide A**

Suresh Kumar[1], Rajiv Dahiya[2*], Sukhbir Lal Khokra[1], Rita Mourya[3], Suresh V. Chennupati[4] and Sandeep Maharaj[2]

[1]*Institute of Pharmaceutical Sciences, Kurukshetra University,*
*Kurukshetra, Haryana, India*
*sureshmpharma@rediffmail.com; slkhokra@kuk.ac.in*
[2]*Laboratory of Peptide Research and Development, School of Pharmacy,*
*Faculty of Medical Sciences,The University of the West Indies,*
*St. Augustine, Trinidad & Tobago, West Indies;*
*rajiv.dahiya@sta.uwi.edu; sandeep.maharaj@sta.uwi.edu*
[3]*School of Pharmacy, College of Medicine and Health Sciences,*
*University of Gondar, Gondar, Federal Democratic Republic of Ethiopia; ritz_pharma@yahoo.co.in*
[4]*Department of Pharmacy, College of Medical and Health Sciences,*
*Wollega University, Nekemte, Federal Democratic Republic of Ethiopia;*
*sureshchennupati@rediffmail.com*

*Content* *Page*

Figure S1.
Mass spectrum for the *N*-methylated cyclic heptapeptide, cordyheptapeptide A ............2

Figure S2.
$^{13}$C NMR spectrum for the *N*-methylated cyclic heptapeptide, cordyheptapeptide A ...….......3

Table S1.
Various steric and lipophilicity parameters for the *N*-methylated linear and cyclic heptapeptide …………4

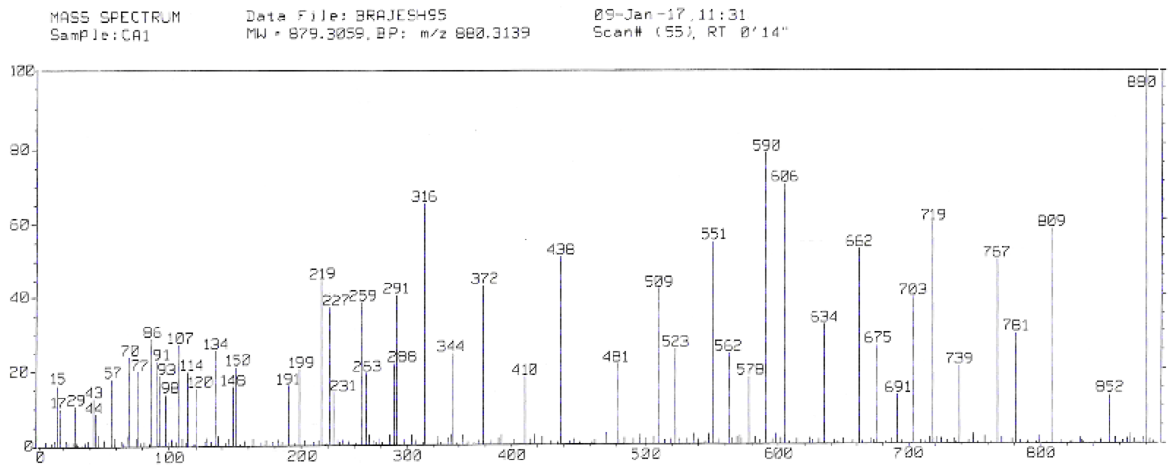


**Figure S1.** Mass spectrum for the *N*-methylated cyclic heptapeptide, cordyheptapeptide A (**8**)

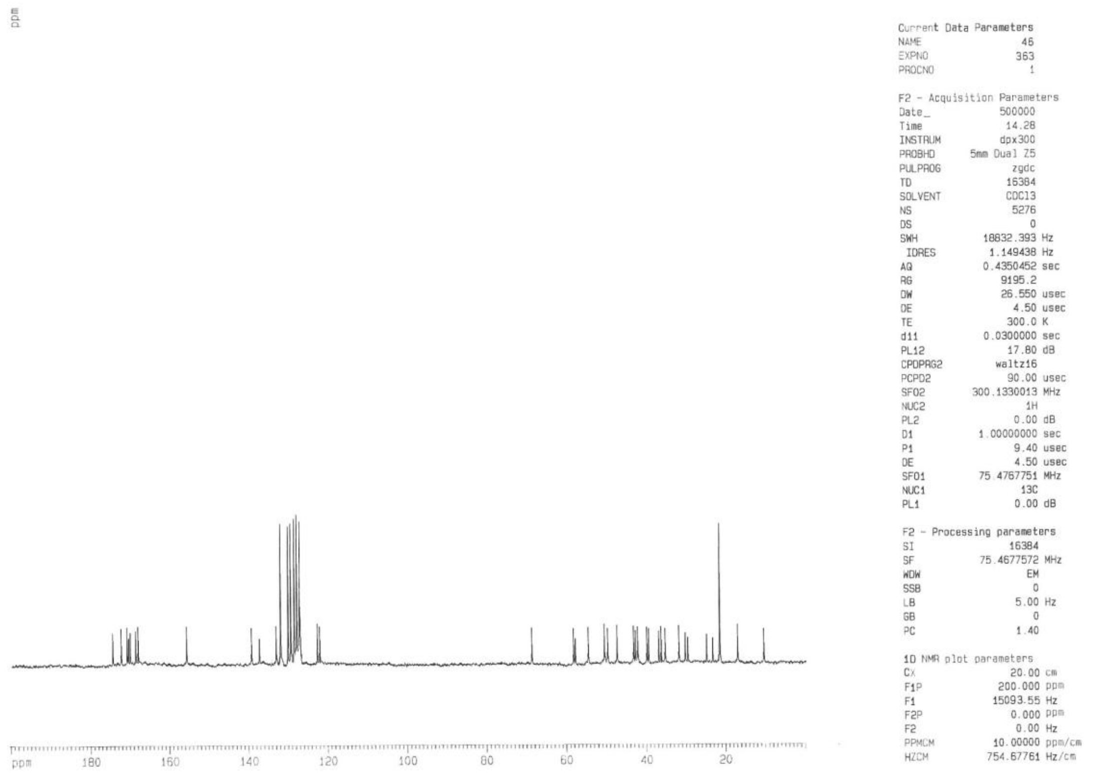


**Figure S2.** $^{13}C$ NMR spectrum for the *N*-methylated cyclic heptapeptide, cordyheptapeptide A (**8**)

**Table S1.** Various steric and lipophilicity parameters for the *N*-methylated linear and cyclic heptapeptide (**7, 8**)

| Parameter | *Calculated value for | |
|---|---|---|
| | **Compound 7** | **Compound 8** |
| Molar Refractivity ($MR^{20}$) | 275.41 ± 0.3 $cm^3$ | 244.28 ± 0.4 $cm^3$ |
| Molar Volume ($MV^{20}$) | 847.8 ± 3.0 $cm^3$ | 697.3 ± 5.0 $cm^3$ |
| Parachor ($P_r$) | 2271.7 ± 6.0 $cm^3$ | 1959.7 ± 6.0 $cm^3$ |
| Refractive Index ($n^{20}$) | 1.563 ± 0.02 | 1.617 ± 0.03 |
| Surface Tension ($\gamma^{20}$) | 51.5 ± 3.0 dyne/cm | 62.3 ± 5.0 dyne/cm |
| Density ($d^{20}$) | 1.193 ± 0.06 g $cm^{-3}$ | 1.26 ± 0.1 g $cm^{-3}$ |
| Polarizability ($\alpha$) | 109.18 ± 0.5 $10^{-24}$ $cm^3$ | 96.84 ± 0.5 $10^{-24}$ $cm^3$ |
| log P (n-Octanol/water) | 8.30 ± 0.89 | 5.43 ± 0.90 |

*Values were calculated using the ACD/ChemSketch 2.0 software
